# Trend of using cementless total knee arthroplasty: a nationwide analysis from 2015 to 2021

**DOI:** 10.1186/s42836-024-00241-7

**Published:** 2024-04-06

**Authors:** Amil R. Agarwal, Emile-Victor Kuyl, Alex Gu, Gregory J. Golladay, Savyasachi C. Thakkar, Gautam Siram, Anthony Unger, Sandesh Rao

**Affiliations:** 1 Department of Orthopaedic Surgery, George Washington University School of Medicine and Health Sciences, Washington, DC 20052 USA; 2 Department of Orthopaedic Surgery, Virginia Commonwealth University, Richmond, VA 23298 USA; 3 Department of Orthopaedic Surgery, Johns Hopkins Medicine, Baltimore, MD 21205 USA; 4 Summit Orthopedics, Washington, DC 20037 USA; 5 Washington Orthopaedics and Sports Medicine, Washington, DC 20006 USA

**Keywords:** Total knee arthroplasty, Cementless fixation, Trends, Utilization

## Abstract

**Background:**

Modern cementless total knee arthroplasty (TKA) fixation has shown comparable long-term outcomes to cemented TKA, but the trend of using cementless TKA remains unclear. This study aimed to investigate the trend of using cementless TKA based on a national database.

**Methods:**

The patients undergoing cementless TKA between 2015 and 2021 were retrospectively extracted from the PearlDiver (Mariner dataset) Database. The annual percentage of cementless TKA was calculated using the following formula: annual number of cementless TKA/annual number of TKA. The trend of the number of patients undergoing cementless TKA was created according to a compounded annual growth rate (CAGR) calculation of annual percentages. Patient age, comorbidity, region, insurance type, etc., were also investigated. Differences were considered statistically significant at *P* < 0.05.

**Results:**

Of the 574,848 patients who received TKA, 546,731 (95%) underwent cemented fixation and 28,117 (5%) underwent cementless fixation. From 2015 to 2021, the use of cementless TKA significantly increased by 242% from 3 to 9% (compounded annual growth rate (CAGR): + 20%; *P* < 0.05). From 2015 to 2021, we observed a CAGR greater than 15% for all age groups (< 50, 50–59, 60–69, 70–74, 75 +), insurance types (cash, commercial, government, Medicare, Medicaid), regions (Midwest, Northeast, South, West), sex (male and female), and certain comorbidities (osteoporosis, diabetes mellitus, tobacco use, underweight (BMI < 18.5), rheumatoid arthritis) (*P* < 0.05 for all). Patients undergoing TKA with chronic kidney disease, prior fragility fractures, and dementia demonstrated a CAGR of + 9%–13% from 2015 to 2021 (*P* < 0.05).

**Conclusion:**

From 2015 to 2021, the use of cementless TKA saw a dramatic increase in all patient populations. However, there is still no consensus on when to cement and in whom. Clinical practice guidelines are needed to ensure safe and effective use of cementless fixation.

## Introduction

Modern innovation in highly porous cementless implants for total knee arthroplasty has reinvigorated the decades-long debate amongst arthroplasty surgeons: to cement or not to cement [1–3]? Although cementless fixation is experiencing a resurgence in popularity due to its improved survivorship, the trend of the number of patients undergoing cementless TKA remains unclear.

Historically, cemented total knee arthroplasty has been the preferred fixation method in most patients, specifically those at high-risk for early implant loosening, such as those with osteoporotic peri-implant bone stock [4, 5]. In addition, the contraindications of cementless TKA included old age (≥ 65 years) and poor bone health. Thus, cemented TKA had remained the gold standard for most patients.

However, fixation failure due to inadequate durability of the bone-cement interface is a major concern, especially in younger patients [6–8]. The three-dimensionally-printed cancellous bone surfaces of cementless designs may provide more physiological and durable fixation [9]. The early studies suggested excellent 5-year survivorship of cementless implants comparable to cemented implants [10–12]. The increasing demand for TKA in younger patients prompts a renewed interest in cementless fixation [13]. With the development of new materials and technologies, the previously established contraindications may be less absolute and more flexible [14, 15]. The purpose of this retrospective study was to analyze the trend of the number of patients undergoing cementless TKA based on a national database between 2015 and 2021. We also analyzed cementless TKA regarding patient age, comorbidity, region, insurance type, etc. We hypothesized that there would be a significantly increasing use of cementless TKA.

## Materials and methods

### Database

We retrospectively reviewed the PearlDiver (Mariner dataset) Database (10435 Marble Creek Circle Colorado Springs, CO 80908, USA). Using the Current Procedure Terminology (CPT) and International Classification of Disease (ICD) 10 billing codes, we identified patients undergoing cementless and cemented TKA from 2015 to 2021. As PearlDiver only releases de-identified patient information to users, the study was deemed exempt from Institutional Review Board approval.

### Patient selection

**Fig. 1:**
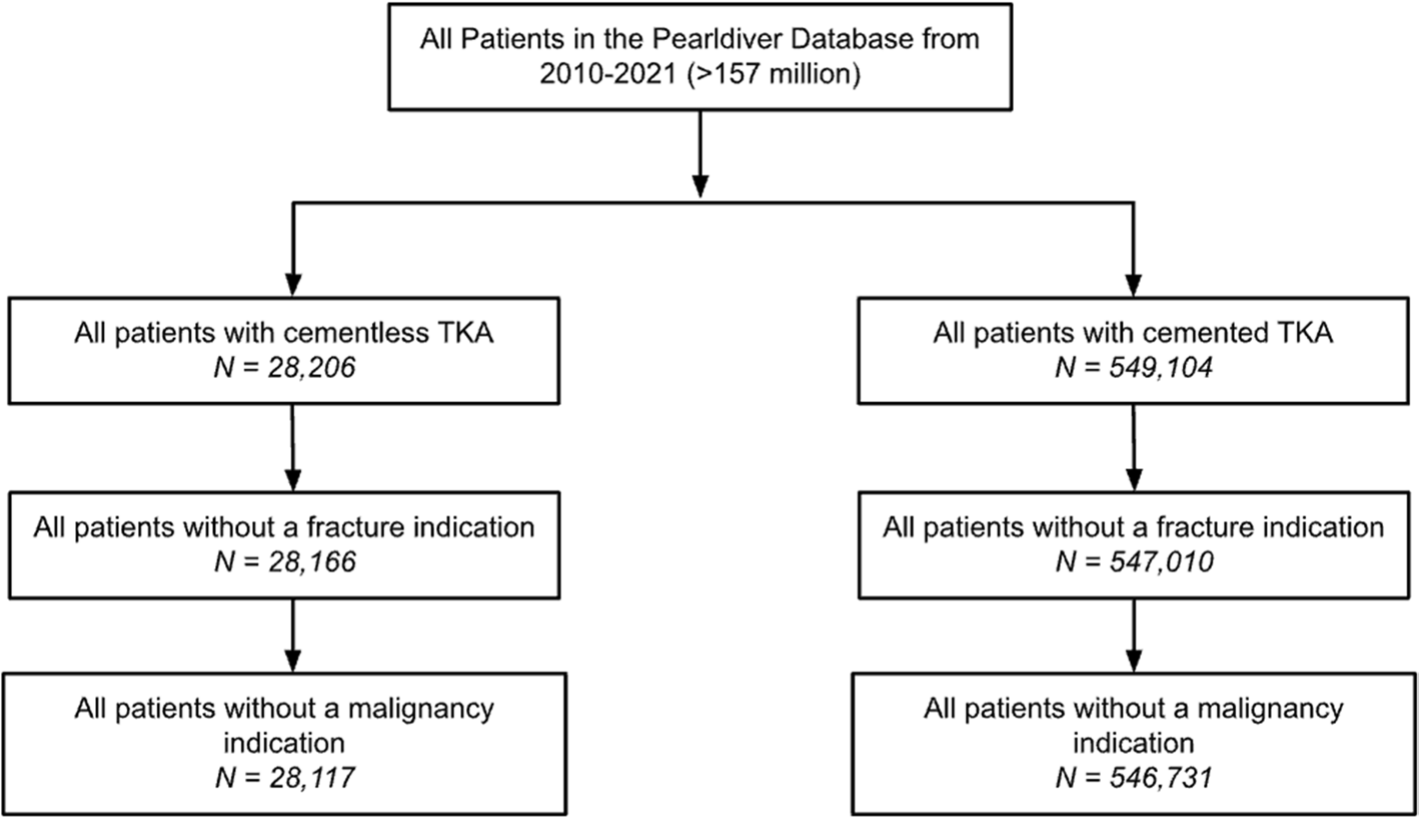
Flow diagram showing inclusion and exclusion criteria

The inclusion criteria of the study included patients between 2015 and 2021 receiving (1) primary cemented TKA (unilateral and bilateral); or (2) primary cementless TKA (unilateral and bilateral); and at least a 2-year follow-up. The exclusion criteria were (1) patients with fracture indications (to only include elective TKA patients); (2) patients with malignancy indications (to only include elective TKA patients) (Fig. 1). In total, 574,848 patients were included in this study with 546,731 (95%) undergoing cemented TKA and 28,117 (5%) undergoing cementless TKA. Table 1 shows the univariate demographics and comorbidities of patients who underwent cemented and cementless TKA.

**Table 1:** Demographics and comorbidities of 574,848 patients undergoing cementless and cemented total knee arthroplasty

Category	Total	Cemented		Cementless		
-	Number	Number	Percentage	Number	Percentage	*P* value
Total	574,848	546,731	95%	28,117	5%	-
Average Age (Years)	-	66.86	SD (9.4)	64.76	SD (9.4)	**< 0.001**
Sex	-	-	-	-	-	**< 0.001**
Women	359,403	343,550	63%	15,853	57%	-
Men	215,443	203,180	38%	12,263	44%	-
Average CCI	N/A	2.1	SD (2.3)	1.97	SD (2.3)	**< 0.001**
Osteoporosis	35,991	34,665	7%	1,326	5%	**< 0.001**
Osteoporosis treatment	48,255	46,433	9%	1,822	7%	** < 0.001**
Prior Fragility Fracture	9,975	9,490	2%	485	2%	0.911
Diabetes Mellitus	37,386	35,578	7%	1,808	7%	0.609
Tobacco Use	15,777	14,890	3%	887	4%	**< 0.001**
Underweight (Body Mass Index < 18.5)	7,260	6,907	2%	353	2%	0.930
Rheumatoid Arthritis	50,055	47,781	9%	2,274	9%	**< 0.001**
Dementia	24,488	23,648	5%	840	3%	**< 0.001**
Chronic Kidney Disease	6,852	6,622	2%	230	0.8%	**< 0.001**
Insurance Type	-	-	-	-	-	**< 0.001**
Cash	1,101	984	0.2%	117	0.4%	-
Commercial	352,115	333,761	61%	18,354	66%	-
Government	7,252	6,817	2%	435	2%	-
Medicaid	21,007	19,714	4%	1,293	5%	-
Medicare	191,738	183,940	34%	7,798	28%	-
Unknown	1,635	1,515	0.3%	120	0.4%	
Region	-	-	-	-	-	**< 0.001**
Midwest	163,699	157,135	29%	6,564	24%	-
Northeast	120,705	113,317	21%	7,388	27%	-
South	199,074	188,496	35%	10,578	38%	-
West	88,662	85,347	16%	3,315	12%	-
Unknown	2,708	2,436	0.4%	272	1%	-

Bolded: *P* < 0.05

*CCI* Charlson Comorbidity Index

### Observation items

We observed patient age (<50, 50–59, 60–69, 70–74, and ≥75), sex (female or male), the Charlson Comorbidity Index (CCI: 0, 1, 2, 3 +), insurance type (cash, Medicare, Medicaid, commercial, Non-Medicare/Medicaid Government Insurance), region (North East, Midwest, South, West), the preoperative diagnosis of osteoporosis, a prior fragility fracture, diabetes mellitus, tobacco use, underweight (body mass index < 18.5), rheumatoid arthritis, dementia, chronic kidney disease, and prior treatment for osteoporosis.

We calculated an annual percentage of cementless TKA using the following formula: annual number of cementless TKA/annual number of TKA. The trend was created according to the annual percentages. We also observed the percentages of cementless TKA in terms of patient age and risk factors.

### Statistical analysis

Compounded annual growth rate was used to determine the rate of change of cementless use from 2015 to 2021 based on the following equation: Compounded annual growth rate = (Y2 value/Y1 value)^1/(Y2 – Y1) – 1^, where Y1 is the first year of the analysis and Y2 is the final year. Compounded annual growth rate (CAGR) is a validated metric of annual change that is commonly used to analyze trends due to its ability to reduce the impact of short-term fluctuations on overall trends [17, 18]. Linear regression analysis was used to observe significant differences in the overall use of cementless TKA as well as the change in patient factors of patients receiving a cementless TKA. *P* values were recorded to show whether there was a significant difference in overall use as well as patient factors, with a *P* value less than 0.05 being statistically significant. With regard to patient selection analysis of those who underwent cemented and cementless TKA, a logistic regression analysis was conducted, reporting the odds ratio (OR), 95% confidence interval (95% CI), and the *P*-value for each variable. All statistical analyses were conducted using R Software (Vienna, Austria) provided by the PearlDiver Database.

## Results

### Patient demographics (univariate analysis)

In total, 574,848 patients were included in this study, with 546,731 (95%) undergoing cemented TKA and 28,117 (5%) undergoing cementless TKA. On univariate analysis, patients undergoing cementless TKA were younger (64.76 ± 9.4 versus 66.86 ± 9.4 years old; *P* < 0.001), more likely to be men (44% vs. 38%; *P* < 0.001), and less likely to have osteoporosis (5% vs. 7%; *P* < 0.001), dementia (3% vs. 5%; *P* < 0.001), and chronic kidney disease (0.8% vs. 2%; *P* < 0.001) when compared to cemented TKA patients (Table 1). The average CCI of cementless TKA patients was significantly lower than the average CCI of cemented TKA patients (1.97 ± 2.3 vs. 2.10 ± ; *P* < 0.001) (Table 1).

### Factors associated with cemented vs. cementless total knee arthroplasty (multivariate analysis)

Following multivariable regression analysis, an age-dependent relationship was observed in those who underwent cementless when compared to cemented TKA. With patients younger than 55 as the reference, those aged 55 to 64 (Odds ratio: 0.90; 95% Confidence interval: 0.87–0.94), 65 to 74 (Odds ratio: 0.73; 95% Confidence interval: 0.70–0.75) and 75 + (Odds ratio [OR]: 0.57; 95% Confidence interval [CI]: 0.50–0.60) were significantly less likely to undergo cementless TKA (*P* < 0.001 for all; Table 2). Female patients (OR: 0.76 times; 95% CI: 0.74–0.78), those with osteoporosis (OR: 0.86; 95% CI: 0.81–0.91), those with dementia (OR: 0.84; 95% CI: 0.79–0.90), and those with chronic kidney disease (OR: 0.73; 95% CI: 0.64–0.83) were also significantly less likely to undergo cementless TKA (*P* < 0.001 for all; Table 2).

**Table 2:** Multivariable analysis for 28,117 patients undergoing cementless total knee arthroplasty

Category	Odd Ratio	95% CI	*P* value
Age (Years)	-	-	-
< 55	Reference	Reference	Reference
55–64	0.90	0.87–0.94	**< 0.001**
65–74	0.73	0.70–0.75	**< 0.001**
75 +	0.57	0.55–0.60	**< 0.001**
Sex	-	-	-
Women	Reference	Reference	Reference
Men	1.32	1.29–1.35	**< 0.001**
CCI	-	-	-
0	Reference	Reference	Reference
1	0.98	0.97–0.99	**< 0.001**
2	0.85	0.80–0.91	**< 0.001**
3 +	0.71	0.62–0.78	**< 0.001**
Osteoporosis	0.86	0.81–0.91	**< 0.001**
Osteoporosis treatment	0.95	0.91–1.00	0.061
Prior Fragility Fracture	1.07	0.98–1.16	0.160
Diabetes Mellitus	1.01	0.96–1.06	0.834
Tobacco Use	1.07	0.95–1.15	0.061
Underweight (BMI < 18.5)	1.10	0.99–1.21	0.081
Rheumatoid Arthritis	0.96	0.92–1.00	0.063
Dementia	0.84	0.79–0.90	**< 0.001**
Chronic Kidney Disease	0.73	0.64–0.83	**< 0.001**
Insurance Type	-	-	-
Commercial	Reference	Reference	Reference
Cash	2.09	1.74–2.51	**< 0.001**
Government	1.19	1.08–1.30	**< 0.001**
Medicaid	1.30	1.23–1.37	**< 0.001**
Medicare	0.99	0.96–1.02	0.483
Region	-	-	-
Northeast	Reference	Reference	Reference
Midwest	0.63	0.61–0.65	**< 0.001**
South	0.87	0.85–0.90	**< 0.001**
West	0.61	0.59–0.63	**< 0.001**

Bolded: *P* < 0.05

*CI* Confidence Interval, *CCI* Charlson Comorbidity Index, *BMI* Body Mass Index

### Trends in use of cementless TKA

**Fig. 2:**
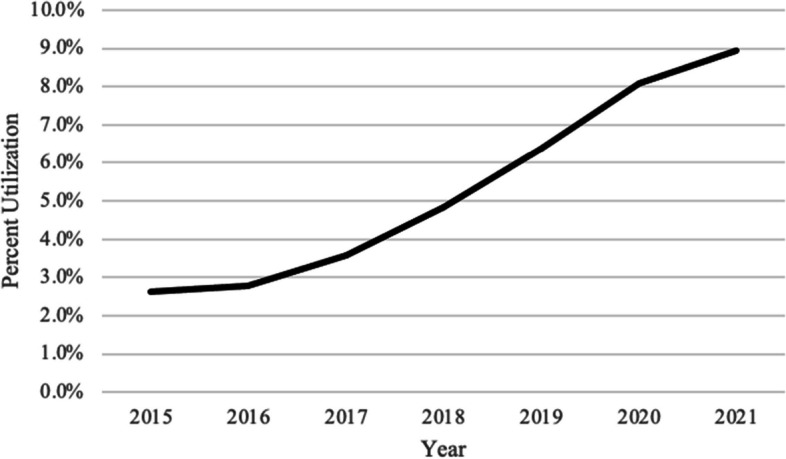
The Trend of the number of patients undergoing cementless total knee arthroplasty between 2015 and 2021

From 2015 to 2021, the use of cementless TKA significantly increased by 242% from 3 to 9% (compounded annual growth rate [CAGR]: +20%; *P* < 0.001; Fig. 2; Table 3). Regarding age, the use of cementless TKA significantly increased for those aged less than 50 (CAGR: +22%, *P* = 0.004), 50–59 (CAGR: +22%, *P* = 0.004), 60–69 (CAGR: +25%, *P* = 0.006), 70–74 (CAGR: +24%, *P* = 0.005), and 75 + (CAGR: +16%; *P* = 0.006; Table 3). The use of cementless fixation significantly increased in men (CAGR: +23%; *P* = 0.005) and women (CAGR: +20%; *P* = 0.005; Table 3).

**Table 3:** Subgroup analysis for the trends of the numbers of patients undergoing cementless total knee arthroplasty

Year	2015	2016	2017	2018	2019	2020	2021	CAGR	*P* value
Total Number	1,025	3,772	4,562	5,477	7,049	4,956	1,276	-	-
Total Percentage	3%	3%	4%	5%	7%	9%	9%	+ 20%	**0.003**
Age (Years)	-	-	-	-	-	-	-	-	-
< 50	5%	6%	7%	9%	11%	13%	17%	+ 22%	**0.004**
50–59	4%	4%	6%	7%	9%	12%	13%	+ 22%	**0.004**
60–69	3%	3%	4%	6%	8%	10%	11%	+ 25%	**0.006**
70–74	3%	3%	3%	5%	6%	8%	10%	+ 24%	**0.005**
75 +	3%	2%	3%	4%	5%	7%	7%	+ 16%	**0.006**
Sex	-	-	-	-	-	-	-	-	-
Women	3%	3%	4%	5%	6%	8%	9%	+ 20%	**0.005**
Men	3%	4%	5%	6%	8%	11%	12%	+ 23%	**0.005**
CCI	-	-	-	-	-	-	-	-	-
0	3%	3%	4%	6%	7%	6%	10%	+ 19%	**0.004**
1	3%	3%	4%	6%	8%	10%	10%	+ 22%	**0.005**
2	3%	3%	4%	5%	7%	9%	11%	+ 25%	**0.007**
3 +	3%	3%	4%	5%	7%	9%	10%	+ 19%	**0.004**
Osteoporosis	3%	3%	3%	4%	6%	7%	7%	+ 17%	**0.004**
Osteoporosis treatment	3%	3%	3%	4%	5%	7%	7%	+ 13%	**0.002**
Prior Fragility Fracture	8%	7%	8%	11%	14%	16%	18%	+ 13%	**0.003**
Diabetes Mellitus	3%	3%	4%	6%	7%	9%	10%	+ 19%	**0.004**
Tobacco Use	3%	3%	5%	6%	8%	9%	14%	+ 26%	**0.009**
Underweight (BMI < 18.5)	2%	3%	5%	5%	7%	8%	9%	+ 27%	**0.004**
Rheumatoid Arthritis	3%	3%	4%	5%	7%	8%	10%	+ 19%	**0.004**
Dementia	3%	2%	3%	5%	5%	7%	6%	+ 11%	**0.004**
Chronic Kidney Disease	4%	3%	3%	4%	5%	6%	6%	+ 9%	**0.001**
Insurance	-	-	-	-	-	-	-	-	-
Cash	5%	8%	10%	13%	14%	17%	24%	+ 27%	**0.004**
Commercial	3%	4%	5%	6%	8%	10%	10%	+ 21%	**0.004**
Government	4%	4%	5%	5%	10%	11%	14%	+ 22%	**0.008**
Medicaid	4%	4%	5%	7%	8%	13%	13%	+ 19%	**0.007**
Medicare	3%	3%	3%	5%	6%	8%	9%	+ 23%	**0.007**
Region	-	-	-	-	-	-	-	-	-
Midwest	3%	3%	4%	5%	6%	7%	9%	+ 20%	**0.004**
Northeast	4%	4%	5%	6%	8%	13%	14%	+ 22%	**0.009**
South	3%	3%	5%	6%	8%	9%	9%	+ 19%	**0.004**
West	3%	3%	3%	4%	6%	8%	9%	+ 23%	**0.004**

Bolded: *P* < 0.05

*CAGR* Compounded Annual Growth Rate, *CCI* Charlson Comorbidity Index, *BMI* Body Mass Index

Regarding comorbidities, the use of cementless fixation significantly increased in those with a CCI of 0 (CAGR: +19%; *P* = 0.004), 1 (CAGR: +22%; *P* = 0.005), 2 (CAGR: +25%; *P* = 0.007), and 3 + (CAGR: +19%; *P* = 0.004; Table 3). The use also increased in those with prior treatment for osteoporosis (CAGR: +13%; *P* = 0.002), those with a prior fragility fracture (CAGR: +13%; *P* = 0.003), those with tobacco use (CAGR: +26%; *P* = 0.009), those who are underweight (CAGR: +27%; *P* = 0.004), and those with a diagnosis of osteoporosis (CAGR: +17%; *P* = 0.004), diabetes mellitus (CAGR: +19%; *P* = 0.004), rheumatoid arthritis (CAGR: +19%; *P* = 0.004), dementia (CAGR: +11%; *P* = 0.001), and chronic kidney disease (CAGR: +9%; *P* = 0.001; Table 3).

Regarding insurance type, the use of cementless fixation increased in those who paid without insurance (CAGR: +27%; *P* = 0.004), those with commercial insurance (CAGR: +21%; *P* = 0.004), those with Medicaid insurance (CAGR: +19%; *P* = 0.008), those with Medicare insurance (CAGR: +23%; *P* = 0.007), and those with non-Medicaid/Medicare insurance (CAGR: +22%; *P* = 0.008; Table 3).

## Discussion

Our study showed an increase in the use of cementless TKA from 3% in 2015 to 9% in 2021. The increased use of cementless TKA persisted in all age ranges (<50, 50–59, 60–69, 70–74, and ≥75), sexes (female and male), CCI (0, 1, 2, and 3 +), insurance types (cash, Medicare, Medicaid, commercial, Non-Medicare/Medicaid Government Insurance), regions (North East, Midwest, South, West), and comorbidities (preoperative diagnosis of osteoporosis, prior fragility fractures, diabetes mellitus, tobacco use, underweight (BMI < 18.5), rheumatoid arthritis, dementia, chronic kidney disease, and prior treatment for osteoporosis).

This increased use is congruent with those reported in national registries. Within this same period, the American Joint Replacement Registry (AJRR) noted an increased use of cementless TKA from 4% in 2015 to 15% in 2021 [16]. Internationally, both the Swedish Knee Arthroplasty Registry and National Joint Registry (covering England, Wales, Northern Ireland, and the Isle of Man and Guernsey) also reported a significantly increased use of cementless fixation, with 8% and 5% of TKA using cementless fixation in 2021, respectively [19, 20]. The higher 2021 reported cementless use in AJRR of 15% when compared to our 9% is most likely due to surgeon-specific factors. Although AJRR is the largest registry of hip and knee replacements in the United States, a higher proportion of contributing surgeons are Hip and Knee Reconstruction fellowship trained orthopaedic surgeons from academic centers when compared to the general population of orthopaedic surgeons [21]. As the PearlDiver database is not a registry where physicians contribute their data but rather a national insurance claims database, it is likely to capture a more generalizable utilization rate of cementless fixation among TKAs performed in the United States.

Consistent with prior findings, this study found younger patients and men were more likely to undergo cementless TKA [10, 22–25]; whereas patients with osteoporosis, chronic kidney disease, and dementia were more likely to undergo cemented TKA [26, 27]. Surgeons’ decision-making regarding fixation modality is highly correlated with bone health. Osteoporosis continues to be a major risk factor for cementless TKA implant failure due to the inherent compromised bone stock and poor potential for bone ingrowth [28, 29]. Therefore, cementation is preferred in this patient population to minimize the risk of periprosthetic fracture [30]. As bone quality is negatively correlated with age, surgeons are more likely to perform cemented TKAs in elderly patients. In addition to osteoporosis, chronic kidney disease (CKD) is highly correlated with postoperative fracture and osteoporosis, prompting surgeons to use cemented fixation [31].

Although younger male patients, without a history of osteoporosis, chronic kidney disease, and dementia are more likely to undergo cementless fixation, our study showed that the increased use of this fixation type still significantly increased in all patient populations assessed from 2015 to 2021. We speculate that the increased use of cementless TKA could be attributed to its cost-effectiveness and lower postoperative complications while maintaining comparable implant survivorship when compared to cemented TKA. In a recent randomized control trial by Tanariyakul et al., cementless TKA was found to have similar functional outcomes and recovery patterns to cemented TKA at a 2-year follow-up [32]. Other randomized control trials have found little to no difference in implant migration or survivorship and clinical, radiographic, or patient-reported outcomes between cementless and cemented TKA components at a 5-, 6-, and 10-year follow-up [1, 3, 33–37]. In a database study by Stavrakis et al. done in 2022, a large cohort of 6,415 cementless TKA patients from 2015 to 2019 was found to have no difference in aseptic loosening when compared with a matched cemented TKA cohort at 90-day, 1-, and 2-year follow-up [27]. However, the authors did find a greater rate of periprosthetic joint infections and fractures in the cementless TKA cohort. From mid- to long-term follow-up, implant survivorship for cementless TKA ranged from 100% at 6-years to 96–99.6% at 10-years, indicating an excellent prognosis that is similar to cemented TKA [34–36]. A meta-analysis by Zhou et al. found no significant differences in implant survivorship and clinical outcomes between both fixation modalities [38]. Prasad et al.’s more recent meta-analysis in 2020 confirmed these findings, showing cementless fixation to be as efficacious as cemented one with an average of 8-year follow-up [39]. Although similarly efficacious, modern cementless fixation may be more cost-effective than cemented ones [40, 41]. While the cost of cementless implants is generally higher than cemented ones, the cost of implanting, considering the cost of cement and operative time, was found to be lower with cementless fixation [40].

As cementless fixation historically has been indicated in younger patients, it is unsurprising that the increased use of cementless fixation is congruent in the younger population. However, our analysis also observed a significantly increased use in the elderly. The emergence of newer bone-preserving implant designs and highly porous metals has permitted the expansion of cementless fixation to older patients [10, 22, 24]. With an average of 4-year follow-up, Goh et al. were among the first to recognize that elderly patients with cementless implants achieve similar patient-reported outcomes and survivorship to those with cemented implants in TKA, explaining the increased use in this patient population [14]. As these implants have been shown to be safe in older patients with most likely lower-quality bone, it stands to reason that this safety profile is congruent in patients with osteoporosis and those at high-risk, as shown by our study’s increased use in these patients. However, long-term implant survivability in these sub-populations has yet to be observed, warranting future works. Additionally, our study found an increased use of cementless fixation regardless of CCI score. CCI is mostly based on medical comorbidities, such as coronary artery diseases, with many not related to bone health. The expansion to “sicker” patients suggests that surgeons may be more comfortable performing this fixation, sticking to bone health factors as their main determinant of fixation type. Lastly, as there are no strict utilization guidelines, there is always the potential for disparities in access. Reassuringly, our results showed increased use of cementless fixation in all insurance types, a known surrogate for social deprivation [42].

The results of this study should be interpreted with respect to its limitations. First, the study was limited to the use of retrospective patient information. We could only report on trends observed and must practice caution in overinterpreting significant results as causation. Second, our analysis was limited to variables provided by the database and was unable to look at surgeon-, hospital-, or implant-specific factors. Third, participating institutions in this nationwide database may have different methods or practices in reporting variables and thus there was the risk of selection biases. Lastly, although we were able to observe that certain patient populations were more or less likely to undergo cementless fixation, we are unable to extrapolate in which patient population cementless fixation should be performed. We observed a universally increased use of cementless TKA across many patient demographics, but surgeons should be aware of some recently reported failures of common cementless implants and avoid their use in high-risk patients [43, 44]. As modern day cementless implants and coatings continue to revolutionize total knee arthroplasty, future studies and clinical practice guidelines should be updated to ensure safe and effective use of cementless fixation.

## Conclusion

From 2015 to 2021, the usage of cementless TKA witnessed a dramatic increase in all patient populations, underscoring an almost universal increase in popularity with limited clarity on when to cement and in whom. Future prospective studies as well as clinical practice guidelines should explore various patient populations to uncover who should undergo cementless fixation based on long-term implant survivorship and incidence of postoperative complications.

## Data Availability

The data that support the findings of this study are available from the PearlDiver database, but restrictions apply to the availability of these data, which were used under license for the current study, and so are not publicly available. Data are however available from the authors upon reasonable request and with permission of the PearlDiver database.

## Abbreviations

TKA: Total Knee Arthroplasty
CCI: Charlson Comorbidity Index
AJRR: American Joint Replacement Registry
CKD: Chronic Kidney Disease
CAGR: Compounded Annual Growth Rate
OR: Odds Ratio
CI: Confidence Interval
